# Overexpression of sonic hedgehog in the triple negative breast cancer: clinicopathological characteristics of high burden breast cancer patients from Bangladesh

**DOI:** 10.1038/srep18830

**Published:** 2016-01-05

**Authors:** A. S. Noman, M. Uddin, M. Z. Rahman, M. J. Nayeem, S. S. Alam, Z. Khatun, M. Wahiduzzaman, A. Sultana, M. L. Rahman, M. Y. Ali, D. Barua, I. Ahmed, M. S. Islam, A. Aboussekhra, H. Yeger, W. A. Farhat, S. S. Islam

**Affiliations:** 1 Department of Biochemistry and Molecular Biology, University of Chittagong, Chittagong, Bangladesh; 2 The Centre for Applied Genomics, The Hospital for Sick Children, Toronto, ON, Canada; 3 Department of Pathology, Chittagong Medical College Hospital, Chittagong, Bangladesh; 4 Department of Genetic Engineering and Biotechnology, University of Chittagong, Chittagong, Bangladesh; 5 Cancer Biology and Experimental Therapeutic Section, Division of Molecular Oncology, King Faisal Specialist Hospital and Research Centre, Riyadh, KSA; 6 Developmental and Stem Cell Biology, Research Institute, The Hospital for Sick Children, Toronto, ON, Canada

## Abstract

Dysregulation of Hedgehog (Hh) signaling pathway has been documented in mammary gland development and breast cancer (BC) progression. Despite the remarkable progress in therapeutic interventions, BC related mortality in Bangladesh increased in the last decade. Triple negative breast cancer (TNBC) still presents a critical therapeutic challenge. Thus effective targeted therapy is urgently needed. In this study, we report the clinicopathological characteristics and prognosis of BC patients from Bangladesh. Routine immunohistochemical analysis and high throughput RNA-Seq data from the TCGA library were used to analyze the expression pattern and association of high and low level of Shh expression in a collection of BC patients with a long-term follow-up. High levels of Shh were observed in a subset of BC tumors with poor prognostic pathological features. Higher level of Shh expression correlated with a significantly poorer overall survival of patients compared with patients whose tumors expressed a low level of Shh. These data support the contention that Shh could be a novel biomarker for breast cancer that is involved in mediating the aggressive phenotype of BC. We propose that BC patients exhibiting a higher level of Shh expression, representing a subset of BC patients, would be amenable to Shh targeted therapy.

Breast cancer (BC) is a heterogeneous disease and has the highest mortality rate in women worldwide. Globally, breast cancer mortality has increased steadily in the last decade. There are about 1.5 million breast cancer patients globally and 200,000 breast cancer patients are added every year. With improved methods in early detection and treatment modalities, the survival rate of patients with breast cancer has increased in the past decade in the industrialized countries^1^. However these trends have not been observed in underdeveloped countries that experience a higher breast cancer incidence and higher mortality rates^2^. Breast cancer related mortality in Bangladesh has steadily increased over the last decade. Approximately 150,000 people die every year in Bangladesh in cancer related disease, and breast cancer related death is top among the list. There is no published data on the actual number of breast cancer patients in the literature showing the clinicopatholgical characteristics of breast cancer in the region, however we estimate an annual breast cancer case burden of approximately 30,000–35,000 cases (data from unsolicited source). We therefore aimed to study the clinicopathological and prognostic characteristics of breast cancer patients from Bangladesh.

Gene expression analysis identified several molecular subtypes of breast cancer that are biologically and clinically distinct^3^. One of these subtypes is triple-negative breast cancer (TNBC), which is negative in estrogen receptor (ER) and progesterone receptor (PR) and also negative in human epidermal growth factor receptor 2 (HER2) overexpression^3^. TNBC accounts for 10–20% of the total breast cancer cases and exhibits a more aggressive clinical outcome and poorer prognosis than other breast cancer subtypes^4^. Although radio, chemo and hormonal therapies have made significant advances in breast cancer treatment, about 20–30% of patients with early detectable breast cancer still experience recurrence with distant metastasis. Moreover, TNBC subtypes usually failed standard adjuvant therapy. In recent years therapeutic targeting of the estrogen receptor (ER: tamoxifen) and Her2 (trustazumab), in ER+ and Her2+ enriched subtypes have shown remarkable improvements with breast cancer therapy. However the majority of TNBC cases that were negative for ER, PR and Her2 usually failed standard adjuvant therapy. Despite progress in early detection and adjuvant chemotherapy the response in women with locally advanced and metastatic disease remains unfavorable outcome [reviewed in^5^]. Therefore, a deeper understanding of the molecular pathways involved in breast cancer progression would lead to more effective targeted therapies to combat TNBC drug resistance and subsequent patient death.

Hedgehog (Hh) signaling pathway is an evolutionary conserved pathway and consists of three ligands, Sonic hedgehog (Shh), Indian hedgehog (Ihh) and Desert hedgehog (Dhh) [ref:^6^). The Shh pathway regulates cell proliferation and differentiation during normal growth and embryonic development and as well as mouse mammary gland development^6^. Shh signaling is mediated by two transmembrane proteins, Smoothened (Smo) and Patched (Patch) and downstream by Gli family transcription factors^7^. The relief of Smo inhibition leads to an activation of Gli family members. Activated Gli1 is then nuclear localized and transcriptionally controls hedgehog (Hh) target genes^8^,^9^. An increased expression of Shh and Gli1 in human breast cancer supports the notion that dysregulation in Hh signaling promotes breast cancer development and progression. The potential role of Shh in breast cancer is not well defined, however recent studies have begun to shed light on its potential importance particularly in aggressive TNBC subgroups^10^,^11^. Recently, we have also shown that Shh overexpression correlates with patient pathological features. We showed that TGF-β1 induced Shh overexpression in bladder cancer and induced Shh promotes bladder cancer invasion and stemness via epithelial-to-mesenchymal transition [EMT]^12^. Hh pathway activation also correlates with a younger age of diagnosis, a high proliferative Ki-67 index, larger tumor size, invasion, lymph nodes metastasis and poor overall survival^13^. Our study and together with others, further confirms that Hh signaling and Hh signaling ligand Shh play a critical role in tumorigenesis.

Although Shh is known to be involved in mammary tumorigenesis and poor survival outcome, more evidence is required in order to fully understand the roles of Shh signaling in breast cancer. The aim of this study was to evaluate the prognostic value of Shh in breast cancer with special emphasize on TNBC. Data from a population based cancer registry (Department of Pathology, Chittagong Medical College and Hospital, Chittagong, Bangladesh) were analyzed. Association of Shh with clinical and histopathologic parameters was evaluated. We report that Shh, a ligand of Hh signaling, is highly expressed in a significant fraction of human breast cancer patients exhibiting a significantly lower overall survival (OS) of the TNBC subtype.

## Patients and Methods

### Patients and clinical information

This study was approved by the ethics committee of the University of Chittagong and Chittagong Medical Hospital, Chittagong, Bangladesh, and was performed in accordance with the ethical standards laid down in the 1964 declaration of Helsinki and all subsequent revisions. Informed consent was obtained from all person mentioned in this paper for the inclusion purposes. Written informed consent was obtained from the patients. Data were prospectively collected on 400 primary female breast cancer patients who underwent treatment/surgery at the Chittagong Medical College and Hospital between September 2005 and January 2013. Formalin fixed paraffin embedded (FFPE) tissues and patients clinical data were collected and compiled retrospectively. Inclusion criteria were women 1) who had unilateral breast cancer, received mastectomy or breast-conserving surgery between the period mentioned above; 2) showed one or more axillary lymph nodes positive for cancer cells by pathologic examination; 3) had no severe concomitant diseases; and^4^ had complete immunohistochemistry data, including estrogen receptor (ER), progesterone receptor (PR) and Her2. The American Joint Committee on Cancer (AJCC, 7^th^ Ed) criteria was followed for the determination of TNM staging of breast cancer. Histological grade was categorized as grade I, II and III following the Nottingham combined histology grading scale conducted by three blinded experienced pathologists. ER/PR and Her2/neu pathological results were obtained from the cancer registry having been processed in Chittagong Medical College and Hospital, Department of Pathology. Detailed scoring system and criteria are presented in Table 1. We considered both borderline and strong positive results for the purpose of this study.

### Immunohistochemical determination of Shh expression

The expression level of Shh was determined by immunohistochemical analyses. Briefly, 10% formaldehyde fixed tissues were embedded in paraffin, 5-μm sections cut, adhered to APES coated slides and dried at 60 °C for 2 h. Paraffin sections were dewaxed in xylene and rehydrated in a series of graded concentrations of alcohol. For antigen retrieval slides were immersed in 10 mM citrate buffer solution (pH 6.0) and boiled in a microwave oven for 10 minutes. The slides were then incubated in 3% hydrogen peroxide solution for 15 minutes at 37 °C, washed in phosphate buffer solution (PBS) and incubated with 4% bovine serum albumin (BSA) in PBS for 30 minutes to block non-specific staining. Sections were incubated with primary antibody, goat polyclonal to Shh (Santa Cruz; cat# sc-1194, CA, USA; dilution 1:100,), at 4 °C overnight. Sections were washed in PBS three times and then incubated with peroxidase conjugated secondary antibody for 1 h at room temperature. Peroxidase substrate containing DAB (3, 3′-diaminobenzidine tetrahydrochloride) chromogen was added to the sections for 5 minutes to develop the reaction.

The slides were viewed under the microscope and scored by counting the number of Shh positive cells based on staining intensity versus the total number of cells and calculating the percentage of positive cells (positive cells/total cells in one field/magnification). The overall staining intensity for Shh positive cancer cells was scored on a 0 to 2 scale, where 0 is negative, 1 (<25%) is low expression and 2 (>25%) is high expression. Tissue sections that did not show any positive brown nuclear staining were scored as 0 (negative). A score of 1 (low expression of Shh) was given if the sections showed occasional positive nuclear staining and a total percentage of positive cells between 5–25%. Sections exhibiting >25% positive nuclear staining (high expression of Shh) was assigned a score of 2+.

### Patient follow-up

Whenever possible patient follow-up was recorded from the patient medical records, hospital visits, telephone or mail contacts, and counted from the first day after surgery every 3 months over a period of 3 years, and every 6 months thereafter. Overall survival (OS) was measured from the date of surgery to the date of death from cancer related or cancer unrelated causes or the date of last follow-up.

### RNA-Seq data sources

RNA-Seq data (Illumina HiSeq 2000 RNA sequencing platform) on 881 breast invasive carcinoma (BRCA) tumor samples were downloaded from The Cancer Genome Atlas (TCGA) for this study and applied to our gene expression analysis. Our analysis focused on the sequencing data Reads per Kilobase per Million mapped reads (RPKM) values. All the quality control and data processing were done by TCGA workgroup at Broad Institute. Level 3 RNA-Seq data contains RPKM values^14^, which is widely used for RNA-Seq normalization methods. The RPKM values can be computed by using a formula: RPKM = 10^9^(C/NL), where C is the number of reads mapped to the gene, N is the total number of reads mapped to all genes and L is the length of the genes.

### Western Blot analysis

Whole cell extracts from breast tumors were prepared in the cell lysis buffer, followed by immunobloting as described^12^. In brief, tissues were lysed in ice-cold RIPA buffer containing 1x protease inhibitor cocktail. Protein concentration was determined using Bradford Assay (Bio-Rad, Philadelphia, PA, USA). Proteins were separated by SDS-PAGE and transferred to nitrocellulose membrane (Millipore, Bedford, MA, USA). After blocking with 5% non-fat dry milk in phosphate buffered saline (PBS)-Tween-20 (PBST), the membranes were incubated with primary antibodies: anti-Shh (Rabbit), anti-ptch1 (Rabbit), anti-SMO (Rabbit), anti-Gli1 (Rabbit) and anti-GAPDH (Rabbit). All antibodies were purchased from Santa-Cruz, CA, USA. After over night primary antibody incubation, membranes were washed 3x with PBST and incubated with secondary antibody conjugated with horseradish peroxidase (HRP). Membranes were washed and bands were visualized on X-ray film using an enhanced chemiluminiscence detection system (Thermo Fisher, USA).

### Statistical analysis

All statistical analysis was performed using Graphpad Prism 5.0 statistical software (Graph Pad, La Jolla, CA, USA) and “R” (version: 3.1.3). The Pearson Chi-square and Fisher’s exact tests were used to calculate the association of categorical variables. Kaplan-Meier survival values were calculated for the low and high expression of Shh. Survival between the groups was compared using the log-rank test. Cox proportional hazard ratio regression was used to determine the gene expression level and survival. Bivariate models examining gene expression had an independent effect on survival. P-values <0.05 were considered statistically significant.

## Results

### Shh overexpression and clinicopathological characteristics of the breast tumor patients

Samples from 400 female breast cancer patients who underwent treatment in Chittagong Medical College Hospital and Cancer Treatment Centre, Bangladesh from September 2005 to January 2013 were reviewed and studied. The average age of the patients was 48.8 (48.88 ± 10.76) years old in a range from 20 to 80 years. The average tumor size was 18.93 mm (SD+/−3.8 mm). Most patients were postmenopausal (63.25%) and presented with tumors at stages pT2 and pT3 (46.0% and 28.0%). The majority of the tumors were Grade II (n = 264; 66.0%) and Grade III (n = 85; 21.25%). Detailed clinicopathological characteristics of the patients’ tumors are shown in Table 2 and Supplementary Table S1.

**Figure 1:**
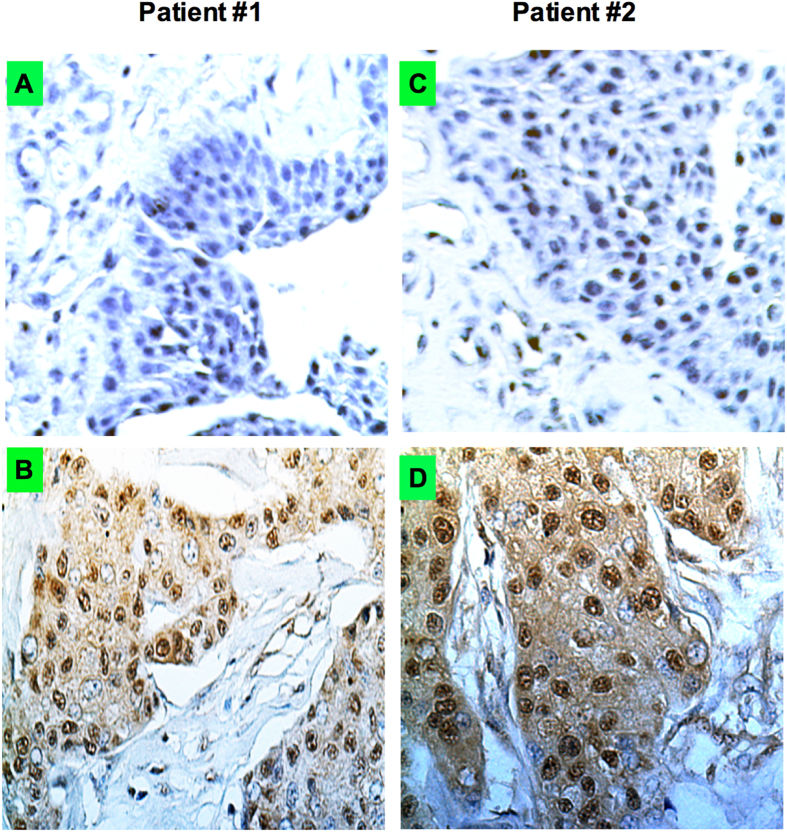
Breast cancers express Shh and expression varies within the tumor. The immunostaining show the expression levels of Shh (X40 magnification). (**A**) Normal breast and (**B**) breast tumor from patient #1; (**C**) normal and (**D**) breast tumor sample from patient #2 show Shh expression. To quantify the Shh expression levels five random areas over each tumor was determined. Representative examples from two patients are displayed here.

To evaluate the expression of Shh in human breast cancer, we used routine immunohistochemical staining (IHS) for Shh protein. Representative samples from two breast cancer patients and a normal breast counterpart with expression of Shh is shown in Fig. 1. The significance in differences of Shh (n = 400) expression and several other clinicopathological parameters were determined (Table 2). The expression intensity of Shh was scored as negative or weakly positive (5–25%) and strongly positive (>25%) [As described in Materials & Methods]. Of the 400 breast cancer samples, 228 (57.0%) patients revealed Shh overexpression (>25%) and 172 (43%) patients were negative or weakly positive (<25%). Most postmenopausal patients were more prone to exhibiting higher Shh values. 89 out of 184 patients with pT2 stage cancer (n = 184; 46.0%) were associated with higher Shh (n = 89; 22.25%; p-0.0040). A statistical significance was found between tumor pathologic grading (p = 0.0384). There were 121 (30.25%) Shh positive tumors among 264 grade II cases that revealed higher Shh protein overexpression. In contrast, 24 out of 85 grade III cases (6.0%) showed Shh overexpression. These data suggest that advanced stage/grade tumors were more likely to overexpress Shh protein than stage pT1/grade I tumors. Furthermore, immunohistochemical analysis revealed that 211/389 (54.24%) were positive for ER, 187/290 (64.48%) were positive for PR and 103/220 (46.82%) were positive for HER2/neu. The expression of Shh (p = 0.0384; 0.0001; 0.0001) was positively correlated with histological grade, ER and PR. A total of 113 (28.25%) patients were found in the TNBC subgroup with higher expression (64; 16.0%; p-0.0001) of Shh protein. Shh overexpression was positively correlated with distant metastasis (p = 0.0001, 75/400, 18.75%). Finally, a total of 52 cases (13.0%; p-0.0035) were found to have locally recurrence associated with high Shh expression in contrast to 35 (8.75%) with low Shh expression group (Table 2; Supplementary Table S1).

### High expression of Shh show poor predicted mortality in breast cancer patients

**Figure 2:**
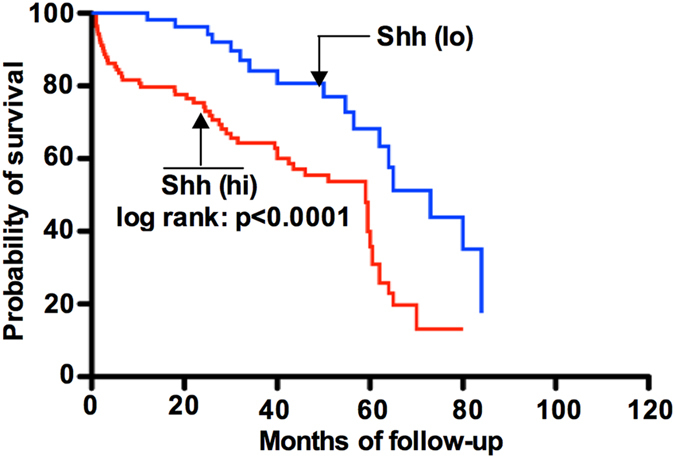
Kaplan-Meier survival curves showing relationship between high and low levels in Shh expression and overall survival in patients with breast cancer. Breast cancer patients expressing a high level of Shh show significantly shorter survival (p = 0.0001) compared to patients expressing a low level of Shh.

We next evaluated association between expression of Shh and overall patient survival (OPS) of all tumor samples. Using Kaplan-Meier analysis where tumors were grouped in high (>25) and low (<25%) level for Shh and a statistical significance was observed between high and low level Shh expressions. Tumors expressing high levels of Shh at levels >25% (Hazard ratio 1.34, p = 0.002, Table 2) is more likely unfavourable prognosis than tumor expressing Shh at levels of <25% (Hazard ratio 0.92; Table 3). Based on the analysis of high and low Shh expression we then analyzed OS of patients. Our results showed that tumors expressing a high level of Shh had reduced OS compared with those with a low level of Shh expression (Hazard ratio; 2.29 (95% CI-1.42-3.53); 5-year survival 49% *vs* 73%; median survival 59 months *vs* 73 months, p = 0.001; Table 4, Fig. 2).

We then analyzed if high Shh is an independent predictor of poor overall outcome using univariate and multivariate analysis and with the following prognostic factors: patient age, tumor stage, tumor grade and receptor status. Univariate and multivariate analyses and Cox regression analysis showed that Shh protein overexpression was an independent prognostic marker for overall survival (Table 5). Statistically significant shorter OS was observed for patients with Shh overexpression in age by univariate and multivariate analysis (HR-2.12; p = 0.021, tumor stage (HR- 1.43; 0.002); tumor grade (HR: 1.45; p = 0.002); and receptor status (HR: 1.31; p = 0.004) [Table 5].

**Figure 3:**
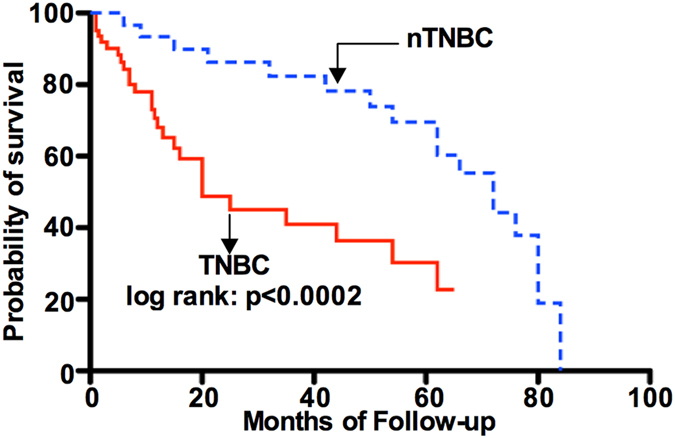
Kaplan-Meier survival curves showing relationship between high and low levels in Shh expression and overall survival in patients with triple negative breast cancer (TNBC). TNBC patients expressing a high level of Shh show significantly reduced survival (p = 0.0002) compared to non-TNBC (nTNBC) patients.

**Figure 4:**
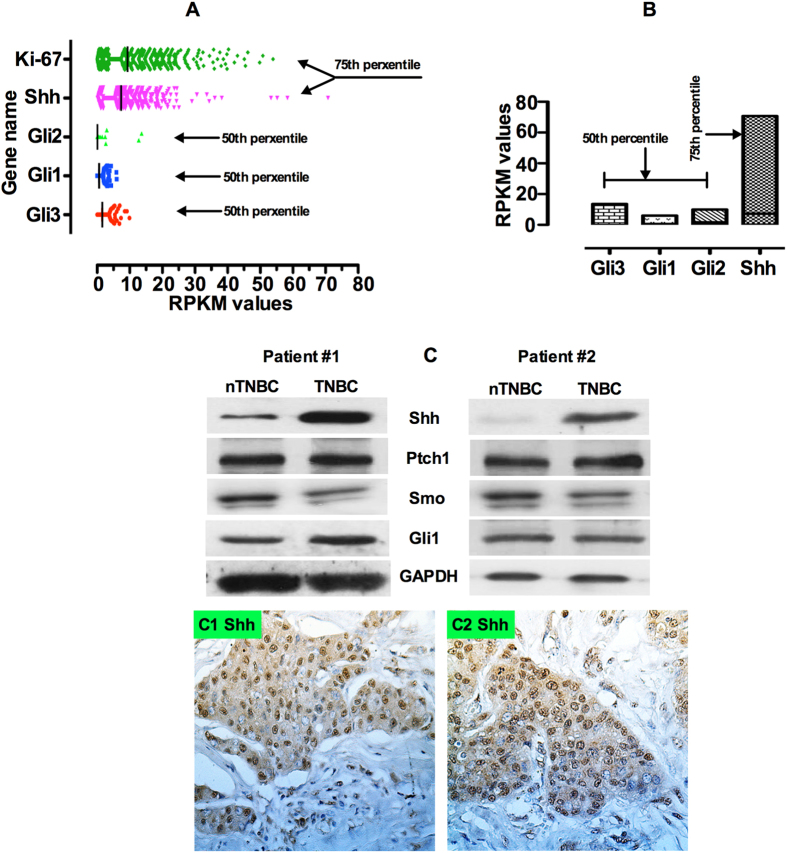
Shh RNA expression in the whole genome in the breast cancers using RNA-Seq library data from a TCGA dataset on 881 patients. (**A**) The figure shows that Shh is highly expressed; Gli1, Gli2 and Gli3 show a moderate or low level of expression for each gene. (**B**) Expression associations between Shh and Gli1, Gli2 and Gli3. (**C**) [Top pane; left and right panel] Western blot analysis results on Shh from two representative triple negative breast cancer patients showing expression intensities of Shh, Ptch1, Smo and Gli1. [Bottom panel; left and right panel] Representative immunostaining from two TNBC patients. To quantify the Shh expression (X40 magnification) levels in five random areas within each tumor were determined.

We next applied bivariate analysis to address factors that might influence the OS in the TNBC subtypes of breast cancer patients. We have carefully chosen and placed emphasis on the TNBC subtype as this subtype shows resistance to drugs, is markedly aggressive and portends poor overall patient survival^14^. Furthermore, the TNBC subtype exhibits more cancer stem cell (CSCs) like characteristics and a higher recurrence rate^15^. Of patients’ tumors, 113 patients were identified in the TNBC subtype group and 64 (15.25%) expressed a high level of Shh. We calculated the OS probability of TNBC based on IHC results and compared with those in the non-TNBC (nTNBC) group. Table 6 show the bivariate analysis of factors associated with OS for the patients with the TNBC subtype. We found that patients with TNBC tumors expressing a high level of Shh had a reduced OS, compared to nTNBC (HR: 3.29, p = 0.002, 5-year survival: 40% *vs* 65%, median survival 20 months *vs* 72 months). We further demonstrated that a high level of Shh expression is an independent predictor of poor OS in bivariate analysis with the other prognostic factors; patient age, metastasis, tumor stage and tumor grade. Bivariate analysis and Cox regression analysis showed that Shh protein overexpression was an independent prognostic marker for OS (Fig. 3). Statistically significant shorter OS was observed for patients with Shh overexpression and with age (HR: 1.12; p = 0.01), metastasis (HR: 1.22, p = 0.003), tumor stage (HR: 1.32; p = 0.002); tumour grade (HR: 1.13; p = 0.002) [Table 7; refer to Fig. 4].

### Validation and comparison of gene expression levels from TCGA RNA-Seq datasets

Supported by National Cancer Institute (NCI) and National Human Genome Research at the National Institute of Health (NHGRI), TCGA is a massive, comprehensive and collaborative platform to catalogue genomic data for over 20 different types of cancers. Gene expression profiling by RNA-Seq is one of the major components of genomic data collected by TCGA. Amongst all other cancer types, TCGA collected a large quantity of tumor-normal paired breast cancer samples. In this study, we selected and analyzed 20,533 genes from 881 RNA-Seq data (as of December 2014) ascertained as breast cancer samples by the TCGA breast cancer repository. Of 20,533 genes, we quantified the expression pattern on several genes of interest, Shh, Ki-67, Gli1, Gli2 and Gli3. We used the RNA-Seq data that was preprocessed for RPKM values computed for each gene by the TCGA by using Java Script and plotted these by using “R” statistical software. The RPKM value is a widely used method for normalizing RNA-Seq gene expression. The gene level RPKM was pre-computed in the database, and data was preprocessed by normalization with 75^th^ and 50^th^ percentiles computed based on the whole genome transcriptome for the entire dataset. Any gene RPKM value with >75 percentile of the genome was considered a highly expressed gene, and medium expression if the RPKM value fell between the 50^th^ and 75^th^ percentiles of the genome. The expression was considered low if the RPKM fell below the 25^th^ percentile. Figure 4A shows the expression of the gene and RPKM values. In the RNA-Seq analysis we carefully used the Ki-67 gene as a reference. Ki-67 is a well-established prognostic marker and a high level of Ki-67 was found to be associated with unfavorable prognosis in breast cancer^16^,^17^. However, American Society of Clinical Oncology (ASCO) does not yet recommend its use as a routine pathological evaluation. From our analysis we observed that Ki-67 is above the 75^th^ percentile cutoff, which suggests that Ki-67 is an independent prognostic marker for breast cancer. On the other hand Shh expression was observed similar to Ki-67 and Gli1, Gli2 Gli3 expression levels were observed in the 50^th^ percentile (Fig. 4A). We then compared the association between Ki67 with Shh, Gli1, Gli2 and Gli3. A clear expression association was identified between Shh (Fig. 4A). Gli2 and Gli3 showed the highest association (Fig. 4A), however, the expression association for Ki67 and Gli1 showed a poor association (Fig. 4A). Finally we compared the association between Shh and expressions all three Hh related Gli1, Gli2 and Gli3 transcription factors. A strong association was observed between Shh, and Gli3, moderate association for Shh and Gli2 and poor association for Shh and Gli1 (Fig. 4B).

Results obtained from RNA-seq data analysis were confirmed by Western blot analysis and immunostaining. We obtained fresh TNBC tumor tissues after surgery and analyzed these for the expression of Shh, Patched 1(Ptch1), Smoothened (Smo) and Gli1 at the protein level. Analyses of two representative patients are shown here (Table 4C; left panel patient#1; right panel patient #2). For patient #1, representing a sample of an nTNBC, Western blot results showed a moderate level of Shh expression in nTNBC, compared with a significant increased in a TNBC sample. However, the nTNBC tumor sample from patient #2 displayed a lack of Shh expression. In both nTNBC and TNBC patient samples, Ptch1, Smo and Gli1 expression remained significantly higher (Fig. 4C). Immunostaining results from both patients exhibited strong nuclear Shh expression (Fig. 4C1,C2). These results further highlight the selective roles of Shh, potentially importance of intratumoral heterogeneity, and its mediators in the progression of breast tumors.

## Discussion

BC is considered to constitute a heterogeneous group of tumors showing different behaviors, prognosis and response to treatment. Gene expression studies revealed several major subtypes of breast cancer. Clinical data on breast cancer patients in Bangladesh populations are limited. We therefore investigated the clinicopathological characteristics and prognostic indications of breast cancer patients in Bangladesh to identify the possible mechanisms responsible for the characteristic growth and metastasis of breast cancer.

The Hh signaling ligand Shh is expressed in various stages of normal mammary gland development, specifically during mammary epithelial bud formation. Although Hh ligand has been shown to play a critical role in development, recent work has focused on understanding its role in tumorigenesis and cancer progression. Dysregulation of the Hh signaling pathway results in multiple organ defects, such as in brain. Patched 1 (Ptch1) mutation in patients with Gorlin’s syndrome shows early onset of multiple basal cell carcinoma^18^ and medulloblastoma^19^. Aberrant Shh expression has been reported in lung^20^, bladder^21^, colorectal^22^, pancreatic^23^ and prostate^24^ cancer. Shh has been thought to play a critical role in carcinogenesis and metastasis in breast cancers. Previous reports showed higher Shh expression in breast cancer and that the Shh-Gli feedback mechanism contributes to breast cancer development and progression. Elevated level of Gli1 expression in breast cancer was correlated with aggressive behavior of the cancer resulting in higher tumor stages and lymph node status^25^,^11^. Although an association between Shh expression and breast cancer has been documented^10^,^11^, the specific role of Shh, correlation with clinicopathological status and the prognostic significance of Shh protein overexpression in breast cancer has remained unclear.

Our data provides evidence for a relationship between high-level Shh expression and poor overall patient survival in breast cancer. We found that Shh overexpression is statistically significantly correlated with increased onset patient age, between 41 and 80 and possibly frequency, than with patients between 20 and 40 years old. Shh protein expression was also found statistically significantly correlated with patient’s menopausal status, tumor states, tumor grading, and receptor status, such as estrogen, progesterone. There is strong evidence that Shh overexpression is associated with the triple negative HER2/neu negative group and the overt clinicopathological characteristics. In support of the results, immunostaining analysis (Fig. 4C1,C2) showed enhanced expression of Shh in the TNBC subtype. This supports a similar finding by other investigators^10^,^11^ who demonstrated that high Hh/Shh expression was significantly correlated with unfavorable prognostic factors. These results are consistent with most published reports that increased Shh expression is correlated with advanced stage cancers in gastric^26^, prostate^27^, medulloblastoma^28^ and breast (Reviewed by)^29^. These results reinforce our claim that the Shh signaling activation loop is functioning to promote breast tumor formation, progression and proliferation.

In several cancers, Shh was found statistically correlated with increased invasion and metastasis^30^. Higher Shh expression in breast tumor was significantly associated with increased risk of metastasis and breast cancer specific death^10^. Consistent with these results our survey also revealed that pT2; pT3 and grade II (GII) tumors showed higher expression of Shh, and that Shh overexpression was statistically correlated with clinicopathological outcome. These results further implicate Shh contributing to breast tumor growth and metastasis. Our bivariate analysis found that high-level of Shh expression was an independent predictor of poor overall survival. In considering this notion we suggest more investigations are necessary to determine whether Shh could be an independent determinant for breast cancer aggressiveness and mortality. The continuous expression of Shh in breast tumors tested in this study allows us to propose that this pathway is a critical and essential component of breast cancer.

Increasing evidence is emerging about the breast cancer stem cell (BCSCs) self-renewal and role of cancer stem cells (CSCs) in breast cancer progression and drug resistance. There are many reports on the role of Hh signaling in the maintenance of cancer stem cells. In breast cancer, pathway activation of CSCs using Shh and Gli1 or Gli2 expression or inhibition with cyclopamine or siRNA mediated against Gli1 or Gli2 alters the expression of Bmi1, which is a central regulator of self-renewal in normal stem cells and tumorigenic potential *in vitro* and *in vivo*^31^. Pathway activation with Shh ligand resulted in CSCs self-renewal and expansion whereas SMO antagonist cyclopamine or the ligand-neutralizing antibody 5E1 induced terminal differentiation and loss of clonogenic growth potential. The Shh pathway is solely activated in cancer stem cells, but not in all cancer cells, and is capable of initiating and sustaining proliferation, invasion and metastasis^32^. Perhaps the tumor microenvironment or niche may influence the ability of Shh directed CSCs to proliferate, migrate or invade.

RNA- Seq data can provide an alternative way to determine the profiling level of gene expression when grades and stages are not known. Our observation on RNA-Seq data from TCGA library also revealed that Shh was highly expressed in the breast tumors we analyzed. We speculate that only high grades and high stages of tumors express Shh genes in these dataset. The RNA-Seq data analyses described in this study adds clarity to the presence and role of Shh in breast cancer particularly in the TNBC subtype tumor. The strong prognostic implication of the Shh pathway and associated pathway genes along with correlations amongst pathway members emphasizes the important role of Shh in the OS of BC patients. Based on our current study, together with previous published data we conclude that high grade and stage and TNBC predict an overall poor patient survival. We suggest further studies will be required to dissect the role of Shh in breast cancer progression, proliferation and CSCs maintenance. Based on our findings here, we suggest that the Shh pathway is activated in the early stages of cancer to enhance tumor growth and proliferation; however, in the later stages it may function in progression and recurrence.

In conclusion, we demonstrated that Shh protein is upregulated in breast cancer. Expression of this protein was statistically correlated with age and malignant stage. Our data demonstrated that, a group of human breast tumors that can be identified by screening for Shh ligand expression might be amenable to treatment with Hh signaling pathway inhibitors, such as cyclopamine and GDC-0449.

## Additional Information

**How to cite this article**: Noman, A.S. *et al*. Overexpression of sonic hedgehog in the triple negative breast cancer: clinicopathological characteristics of high burden breast cancer patients from Bangladesh. *Sci. Rep*. **6**, 18830; doi: 10.1038/srep18830 (2016).

## Supplementary Material

Supplementary Information

## Figures and Tables

**Table 1:** Methods of scoring system and criteria for determining ER/PR and Her2/Neu positivity and negativity.

The ER/PR scoring methods and criteria	
Scoring methods	
0	Negative for ER/PR receptors
1	Barely detected
2+/3+	Positive for ER/PR receptors
Criteria	
0	No or 0% nuclear staining
1+	<15% or occasional nuclear staining (1–15%)
2+	>15% to 75% clear positive nuclear staining
3+	>75% positive staining
Her2/Neu scoring method and criteria	
0	Negative.
1+	Negative/very weakly positive.
2+	Weakly sporadic positive
3+	Positive
Criteria	
0	0% or no staining was observed, negative staining.
1+	Barely/very faint membranous staining but the staining did not reach more than 10%.
2+	The staining intensity was weak, occasional membranous staining was observed, total positive staining tumor cells higher than 10%.
3+	Strong consistent positive membranous staining in more than 10% of tumor cells.

**Table 2:** Patients characteristics: Association of Shh expression with the clinicopathological outcome of 400 patients with breast cancer.

Characteristics	n = 400	Shh (+) >25% expression	Shh(−) <25% expression	p-values
Age				0.8682/0.9185^a^
<40	167(41.75)	96(24.0)	71(17.75)	
>40	233(58.25)	132(33.0)	101(25.25)	
Menopausal status				0.0021/0.0031^a^
Premenopausal	147(36.75)	79(19.75)	68(17.0)	
Postmenopausal	253(63.25)	146(61.5)	107(26.75)	
Tumor stage				0.0040/0.0040^a^
pT1	76(19.0)	22(5.5)	54(13.5)	
pT2	184(46.0)	89(22.25)	95(23.75)	
pT3	112(28.0)	42(10.5)	70(17.5)	
pT4	28(7.0)	12(3.0)	16(4.0)	
Tumor grade				0.0384/0.0430^a^
G1	51(12.75)	20(5.0)	31(7.75)	
G2	264(66.0)	121(30.25)	143(35.75)	
G3	85(21.25)	24(6.0)	61(15.25)	
Receptors status Estrogen				0.0001/0.0001^a^
Positive	211(54.24)	103(26.47)	108(27.76)	
Negative	178(45.76)	52(13.37)	178(45.76)	
Progesterone				0.0001/0.0001^a^
Positive	187(64.48)	76(26.21)	111(38.28)	
Negative	103(35.52)	12(4.14)	91(31.38)	
HER2/Neu				0.4223/0.4960^a^
Positive	103(46.82)	61(27.73)	42(19.10)	
Negative	117(53.18)	63(63.64)	54(24.55)	
Molecular classification				0.0001/0.0001^a^
Triple negative	113(28.25)	64(16.0)	49(12.25)	
Non-triple negative	287(72.0)	26(6.6)	261(65.25)	
Distant metastasis				0.0001/0.0001^a^
Positive	120(30.0)	75(18.75)	45(11.25)	
Negative	280(70.0)	104(26.0)	176(44.0)	
Local recurrence				0.0035/0.0047^a^
Yes	87(21.75)	52(13.0)	35(8.75)	
No	313(78.25)	163(40.75)	50(37.5)	

Significance level: *p<0.05* (^a^-Fisher Exact Test p-values).

**Table 3:** Cox’s Proportional Hazard Ratio (HR): Survival analysis.

	n	HR (95% CI)	HR>25%	HR<25%	p-value
Shh	400	1.63(1.01–2.04)	1.34(1.02–1.79)	0.92(0.78–1.06)	0.002

**Table 4:** Comparison of tumors expressing high and low levels of Shh in breast tumors.

		n	HR (95% CI)	5-year survival (%)	Median survival (95% CI)	p-values
	High	228		49	59 months (1.10–1.46)	
Shh			2.29(1.42–3.53)			0.001
	Low	172		73	80 months (0.95–1.11)	

**Table 5:** Univariate and multivariate analysis of overall survival in breast cancer patients.

Characteristics	Univariate analysis		Multivariate analysis	
	HR (95% CI)	p value	HR (95% CI)	p values
Age				
<40	1*			
>40	1.50(1.31–2.42)	0.016	1.40(1.41–2.41)	0.003
Shh	2.12(1.45–2.32)	0.021	1.34(1.02–1.78)	0.001
Tumor stage				
pT1	1*		1*	
pT3	2.034(1.87–2.97)	0.002	1.34(0.97–3.32)	0.01
pT4	2.24(2.18–5.23)	0.003	2.01(1.25–5.14)	0.001
pT4	3.41(3.21–6.31)	0.003	2.30(3.21–5.41)	0.02
Shh	1.43(1.21–2.34)	0.002	1.12(1.01–1.76)	0.001
Tumor grade				
G1	1*		1*	
G2	1.50(0.03–2.41)	0.008	1.02(0.31–2.61)	0.006
G3	2.07(1.01–3.28)	0.004	1.21(1.05–2.41)	0.003
Shh	1.45(1.02–1.87)	0.002	1.12(0.98–1.45)	0.005
Receptor status				
Negative	1*		1*	
Positive	1.21(1.01–2.21)	0.006	0.95(0.51–1.12)	0.002
Shh	1.31(1.02–1.62)	0.004	0.94(0.91–1.12)	0.003

1^*^ Grade 1 is regarded as reference category.

**Table 6:** Comparison of tumors expressing high and low levels of Shh in TNBC patients.

		n	HR (95% CI)	5-year survival (%)	Med. survival (95%CI)	p-values
	High	61		40	20 months (1.10–1.46)	
Shh			3.29(1.42–5.53)			0.002
	Low	42		65	72 months (0.95–1.11)	

**Table 7:** Overall survival in TNBC subtype breast cancer.

Characteristics	n	Bivariate analysis	p-values
		HR (95% CI)	
Age	113	1.07(1.03–1.27)	0.01
Shh		1.12(1.03–1.41	0.002
Metastasis	110	3.43(3.56–6.22)	0.02
Shh		1.22(1.02–1.13	0.003
Tumor stage	113		0.001
pT1		1*	
pT2		1.03(0.972–3.32)	
pT3		1.07(1.02–1.27)	
pT4		2.08(1.08–3.17)	
Shh		1.32(1.03–1.25)	0.002
Tumor grade	113		0.001
G1		1*	
G2		1.21(0.99–2.31)	
G3		1.31(1.05–2.23)	
Shh		1.13(1.02–1.27	0.002

1^*^ Grade 1 is regarded as reference category.
